# The establishment of an immunosensor for the detection of SPOP

**DOI:** 10.1038/s41598-021-91944-3

**Published:** 2021-06-15

**Authors:** Song Yue, Kexin Sun, Siyuan Li, Yi Liu, Qihao Zhu, Yiyu Chen, Dong Yuan, Tao Wen, Mingjian Ge, Qiubo Yu

**Affiliations:** 1 Institute of Life Science, Chongqing Medical University, 1 Yi Xue Yuan Road, Chongqing, 400016 People’s Republic of China; 2 Department of Ophthalmology, Chongqing Key Laboratory of Ophthalmology, The First Affiliated Hospital of Chongqing Medical University, Chongqing Eye Institute, Chongqing, 400016 People’s Republic of China; 3 Department of Thoracic Surgery, The First Affiliated Hospital of Chongqing Medical University, Chongqing, 400016 People’s Republic of China

**Keywords:** Cancer, Chemistry, Materials science, Nanoscience and technology

## Abstract

In this paper, we first synthesis three-dimensional jasmine-like Cu@L-aspartic acid(L-ASP) inorganic–organic hybrid nanoflowers to load palladium-platinum nanoparticles (Pd–Pt NPs) as the signal enhancer in order to quantify intracellular speckle-type POZ domain protein. Scanning electron microscope, fourier transform infrared, energy dispersive spectrometer, X-ray photoelectron spectroscopy analysis was used to characterize the newly synthesized materials. The newly formed Cu@L-Asp/Pd-PtNPs can catalyze the decomposition of hydrogen peroxide and exhibit excellent catalytic performance. When different concentration of speckle-type POZ domain protein is captured by speckle-type POZ domain protein antibody linked to the surface of Cu@L-Asp/Pd–Pt NPs, the current signal decreases with the increase concentration of speckle-type POZ domain protein. After optimization, the speckle-type POZ domain protein immunosensor exhibited a good linear response over a concentration range from 0.1–1 ng mL^−1^ with a low detection limit of 19 fg mL^−1^. The proposed sensor demonstrates good stability within 28 days, acceptable reproducibility (RSD = 0.52%) and selectivity to the speckle-type POZ domain protein in the presence of possible interfering substances and has potential application for detecting other intracellular macromolecular substances.

## Introduction

Ovarian cancer is one of the three most common malignant tumors in the female reproductive tract and it is not easy to be detected^1^. About 70% of ovarian cancer patients are in advanced stage and have a low five-year survival rate (20–30%)^2,3^. Speckle-type POZ domain protein(SPOP)is mainly composed of 374 amino acids, whose N-terminal and C-terminal contain a typical POZ/BTB domain and a MATH/TRAF domain respectively^4^. In recent years, researchers have found that SPOP is closely related to tumor proliferation and invasion in the process of studying the bio-ethology of tumors^5–8^. Previous studies of our group have shown that SPOP protein with low expression level can promote proliferation and migration of ovarian cancer cells^9^. Therefore, detection of SPOP in cells is of great value for the cytology study and prognosis of ovarian cancer. Traditional techniques for quantitative detection of protein include high-performance liquid chromatography-mass spectrometric, bioluminescent immunoassay or fluorescence immunoassay^10–13^. However, these methods all have the problems of high cost, low sensitivity and requiring long detection time. Electrochemical immunosensor is a sensor that combine both of the immune technology and electrochemical detection method and the combination of high sensitivity sensing technology and specific immune reaction makes electrochemical immune sensor have good selectivity, high accuracy and wide detection range^14–17^. Moreover, label-free electrochemical immunosensors can detect antigen–antibody binding by monitoring the changes in the electronic or interfacial properties^18^. Based on the above reasons, we first use label-free electrochemical immunosensor to quantitatively detect intracellular SPOP.

Metal nanoparticles are widely used in cancer diagnosis, fuel-cell electrocatalysts, hydrogen-storage materials, and sensors^19–21^. Among them, because of its excellent stability and good catalytic activities, platinum nanoparticles (Pt NPs) has wide range of applications in the biosensors, fuel cells, as well as electrocatalytic oxidation^22^. However, single platinum nanoparticles usually have the disadvantages of high cost and relatively low catalytic activity^23^, therefore, the key issue is how to improve both of the activity and utilization efficiency of Pt NPs.

Compared with monometallic nanoparticles, bimetallic nanoparticles have successfully aroused researchers’ interest since they show good optical, electrical, as well as chemical properties. Moreover, bimetallic nanoparticles also present better catalytic properties than their counterparts for the two metals can create cooperative effects, which signifies the whole being stronger than the sum of its parts^24^. In this paper, Pt NPs and palladium nanoparticles (Pd NPs) are combined to form palladium-platinum bimetallic alloy and because the Pd NPs can alter the electronic structure^25^, the newly formed palladium-platinum nanoparticles (Pd–Pt NPs) show enhanced catalytic activity towards hydrogen peroxide.

Among a wide variety of support nanomaterials that have been used to avoid aggregation between metal nanoparticles, nanomaterials with three-dimensional (3D) nanostructure stand out for much more highly arranged structure than their one-dimensional or two-dimensional counterparts^26^. Besides, 3D structure has higher surface-to-volume ratio so that more attachment sites could be provided to load signal enhancer, further increasing the sensitivity of the developed immunosensor^27^.

Since the first kind of organic–inorganic hybrid nanoflower(HNFs) created by Zare’ group^28^, it has become another commonly used nanocarrier integrated into electrochemical biosensors due to its efficient loading capability, good biocompatibility, powerful capture ability, and high surface-to-volume ratio(which does not lead to significant obstruction of the electron transfer process)^29^. L-Aspartic acid is acidic amino acid that each molecular contains two carboxyl group, wherein carboxyl oxygen could chelate almost all metal ions in oxidation state^30^. Specially, due to the ease of synthesis, outstanding chemical properties, HNFs show promising application in electrochemical biosensing^31^. In this work, we selected L-Aspartic acid as the organic part and copper(II) ions as the inorganic part to synthesize 3D-structure jasmine-like nanoflowers for the first time in order to load more Pd–Pt NPs as well as avoid aggregation between metal nanoparticles. The brand-new Cu@L-Asp/Pd–Pt NPs nanocomposite is easy to synthesize and it can improve the detection sensitivity and limit of detection values.

Herein, we report on a ultrasensitive, innovative label-free immunosensor using the first synthesized Cu@L-Asp hybrid nanoflowers as the supporting materials to load palladium-platinum bimetallic alloy. The process of synthesizing Cu@L-Asp is relatively simpler than that of other composite materials and the catalytic ability of Cu@L-Asp/Pd–Pt NPs is significantly stronger than that of individual Pd NPs, Pt NPs or Pd–Pt NPs in the presence of H_2_O_2_. This proposed label-free immunosensor showed excellent analytical performance for SPOP, indicating its great potential for rapid and accurate SPOP determination.

## Experimental section

### Materials and chemicals

A human SPOP ELISA kit was purchased from Shanghai Jianglai Bio-Technology Co., Ltd (Shanghai, China). Copper chloride (CuCl_2_), L-Aspartic acid (L-Asp), sodium hydroxide (NaOH), hydrochloric acid (HCl), hydrogen peroxide (H_2_O_2_), sodium tetrachloropalladate (II) (Na_2_PdCl_4_), chloroplatinic acid (H_2_PtCl_6_·6H_2_O) was obtained from Aladdin (Shanghai, China). Lysozyme was obtained from Solarbio Life Science (Beijing, China). Glutathione (GSH), Horseradish Peroxidase (HRP), Catalase (CAT) were purchased from Sigma-Aldrich (St Louis, USA). IOSE80 cell line was obtained from Shengzhen Huatuo Bio-Technology Co., Ltd (Shengzhen, China). The other reagents used in this experiment were of analytical grade and all of the water used in the tests was obtained from a Millipore Mill-Q purification system(> 18.2 MΩ cm, USA).

### Apparatus and characterization

In this experiment, we use the conventional three-electrode system to perform the electrode detection tests. A glassy carbon electrode (GCE, 4 mm in diameter) was used as the working electrode, whereas a platinum wire was used as the auxiliary electrode and a saturated calomel electrode (SCE) was used as the reference electrode. The measurement of amperometric i-t curves and the cyclic voltammetry(CV) tests were performed using a CHI660E electrochemical workstation(Shanghai Chenhua Apparatus Corporation, China). The morphologies of the Cu@L-Asp hybrid nanoflowers and Cu@L-Asp/Pd–Pt nanocomposites were analyzed via field emission scanning electron microscopy (SEM, SU8010, Japan). Energy dispersive X-ray spectroscopy (EDS) were carried out using Oxford X-max50 microscope (Oxford England). X-ray photoelectron spectroscopy (XPS) measurements were performed with a Thermo scientific ESCALB 250 Xi spectrometer (Thermoelectricity Instruments, USA). Fourier transform infrared (FT-IR) was tested using a Nicolet 6700 FT-IR spectrometer (Thermo Nicolet, USA).

### Synthesis of the Cu@L-Asp hybrid nanoflowers

**Scheme 1:**
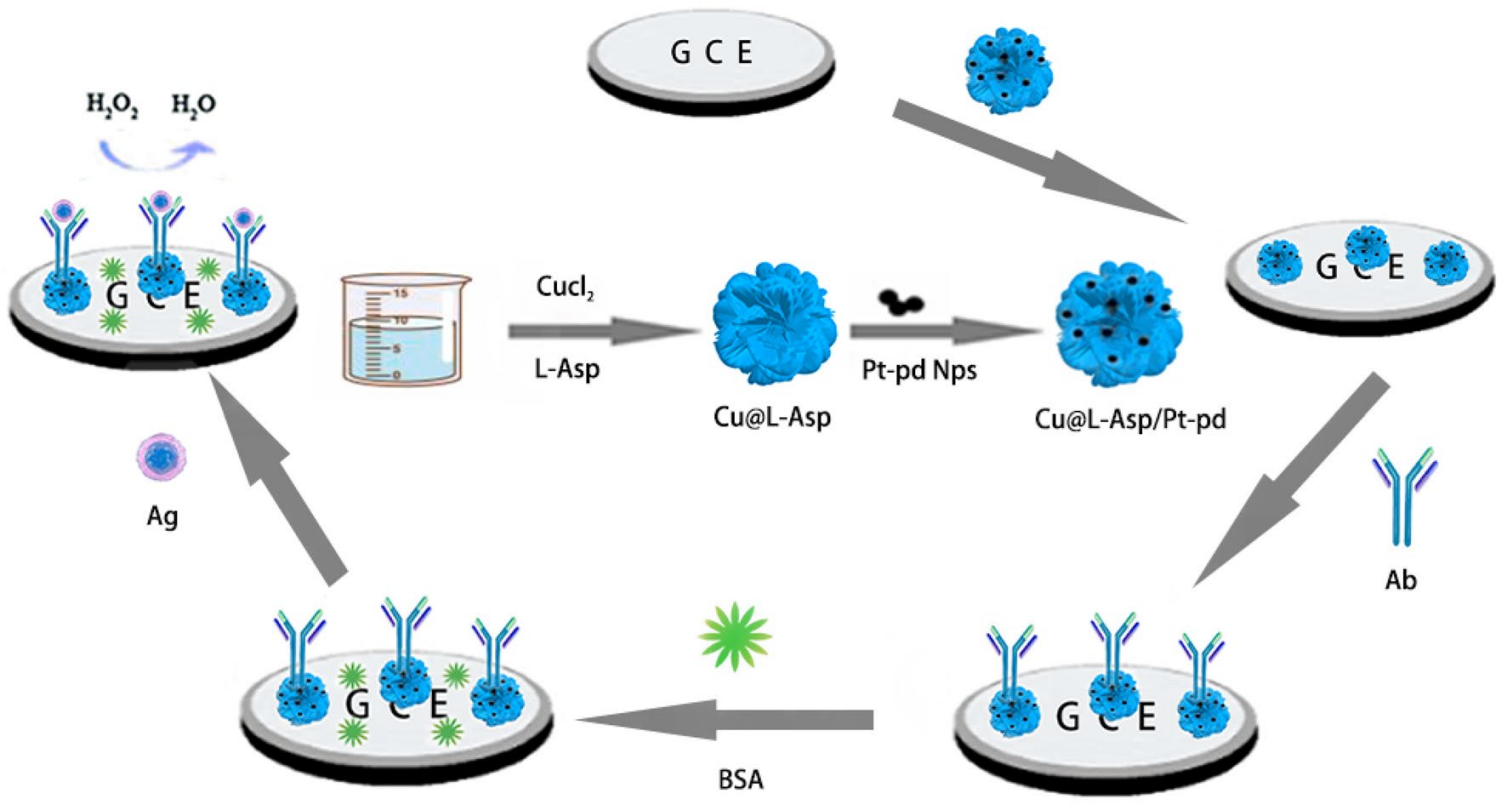
Fabrication process of the proposed label-free electrochemical immunosensor.

To synthesis three-dimensional jasmine-like Cu@L-Asp hybrid nanoflowers, 10 mg CuCl_2_·2H_2_O and 15 mg L-Asp were dispersed in phosphate buffered saline (PBS, 0.1 M) with gently stirring overnight. Afterwards, the mixture was purified by centrifugation and washed with ultrapure water three times. Subsequently, the obtained solution was dispersed in ultrapure water and conserved in a refrigerator(4 °C) for further use. The preparation process of the three-dimensional jasmine-like Cu@L-Asp hybrid nanoflowers is demonstrated in Scheme 1.

### Synthesis of Pd-PtNPs, PdNPs, PtNPs

To achieve signal amplification of the electrochemical immunosensor, Pd-PtNPs were used as a signal enhancer. Pd-PtNPs were synthesis according to the literature with slight modifications^24^. Briefly, 90 µl of H_2_PtCl_6_ (5%) and 119 µl of Na_2_PdCl_4_ (5%) was added to 10 mL of ultrapure water. Afterwards, the mixed solution was added with 10 mL of NaBH_4_ solution (0.28 mg/mL) and then gently stirred for 1 h. Then, the complex solution was centrifuged at 12,000 rpm and washed with ultrapure water and ethanol three times, respectively. Finally, the mixture was dispersed in the 2 mL of ultrapure water for further use. Detailed synthesis methods of palladium and platinum nanoparticles will be provided in supplementary materials.

### Preparation of Cu@L-Asp/Pd–Pt NPs, Cu@L-Asp/Pd NPs, Cu@L-Asp/Pt NPs

Since the prepared Cu @ L-Asp nanoflower has high specific surface area and rich amino group, it could be used as an excellent platform for in-situ assembly of Pt–Pd NPs. We added Cu@L-Asp and Pd–Pt NPs to ultrapure water in a ratio of 1:15 and stirred them continuously at room temperature for 12 h. The resulting solution is then placed in a centrifuge at 3000 RPM for 5 min. Finally, the supernatant is sucked out with a pipette and washed with ultrapure water for three times. The synthetic methods of Cu@L-Asp/Pt NPs and Cu@L-Asp/Pd NPs is similar to that of Cu@L-Asp/Pd–Pt NPs, except that Pd–Pt NPs were displaced by Pd NPs and Pt NPs, respectively.

### Cell thawing

First, the ultraviolet lamp was turned on to irradiate the sterile operating table for 30 min, and meanwhile the culture medium and PBS were placed in a 37℃ water bath box for the next experiment. Next, the sterile operating table was wiped with an alcohol cotton ball for disinfection, and then we prepared a cell culture medium containing 10% serum (10 mL serum + 90 mL PBS) on the sterile operating table. After that, 2 mL of the above culture medium was taken into a centrifuge tube for standby use. The frozen cells were then removed from the − 80℃ refrigerator and quickly placed in a 37℃ water cup for melting and then centrifuged (800 rpm, 4 min). Finally, the well-prepared cells were transferred into a new cell culture bottle containing 3 mL culture medium and incubated overnight.

### Cell subculture

The culture medium was poured out and rinsed with PBS for 3 times (3 mL PBS each time), then 1 mL trypsin was added, shaken from side to side for 15 s, and the digestion was terminated with 2 mL culture medium. After that, the complex solution was centrifuged at 800 rpm for 4 min. Subsequently, the supernatant was discarded and transferred into the new culture bottle. Finally, they were put back to the incubator for the next round.

### Cell lysis

The culture medium was poured out and then washed with PBS (pre-cooled) twice, and the adherent cells were scraped on the ice with cell scraper, then blown evenly with 1 mL PBS, and centrifuged at 1000 rpm 4℃ for 5 min. Subsequently, discard the supernatant and add 100ul RIPA and 1ul PMSF, then centrifuge at 12000 rpm 4℃ for 20 min. Finally, transfer the supernatant into a new EP tube for further use.

### Fabrication of the electrochemical SPOP sensor

The fabrication process of the label-free immunosensor is shown in Scheme 1. First, we use the aluminum oxide powder with a diameter of 0.3 μm to polished the surface of the electrode and each electrode lasted for 5 min. Then ultrasonic cleaning was carried out on the polished electrode in the order of ultra-pure water, ethanol and ultra-pure water respectively, 5 min for each step. Finally, the electrode was polished again with aluminum oxide powder with a diameter of 50 nm in accordance with the above steps. After the electrode was dried, 10μL of the Cu@L-Asp/Pd–Pt NPs nanocomposite solution was added to the surface of the pretreated clean electrode. After the electrodes have dried at room temperature, 6μL of anti-SPOP was dropped onto the electrodes and combined with Cu@L-Asp/Pd–Pt NPs by Pt-NH2 and Pd-NH2 bond and incubated for 4 h at 37 °C. In order to block the nonspecific sites, the electrodes were coated with a BSA solution(1%, w/v) at room temperature for 30 min. Finally, the well-constructed electrodes were conserved in a refrigerator(4 °C) for further use.

### Measurement procedure

Prior to the measurement, we dripped different concentrations of SPOP antigen onto the electrodes that had been constructed and incubated for 1 h at 37 °C and after that, the ultrapure water was used to remove the unbound compounds. Then, the immunosensor were dried at room temperature before the following experiment. The i-t curve was carried out using − 0.4 V as the starting voltage, and 20 μL H_2_O_2_ was added to the PBS (0.1 M,PH = 7.4) after the background current was stable. The change of current was obtained according to the following formula: Δcurrent = current 1- current 0, where current 1 represents the current when different concentration of SPOP and 20 μL H_2_O_2_ was added, and current 0 is the background current.

## Result and discussion

### Choice of materials

**Figure 1:**
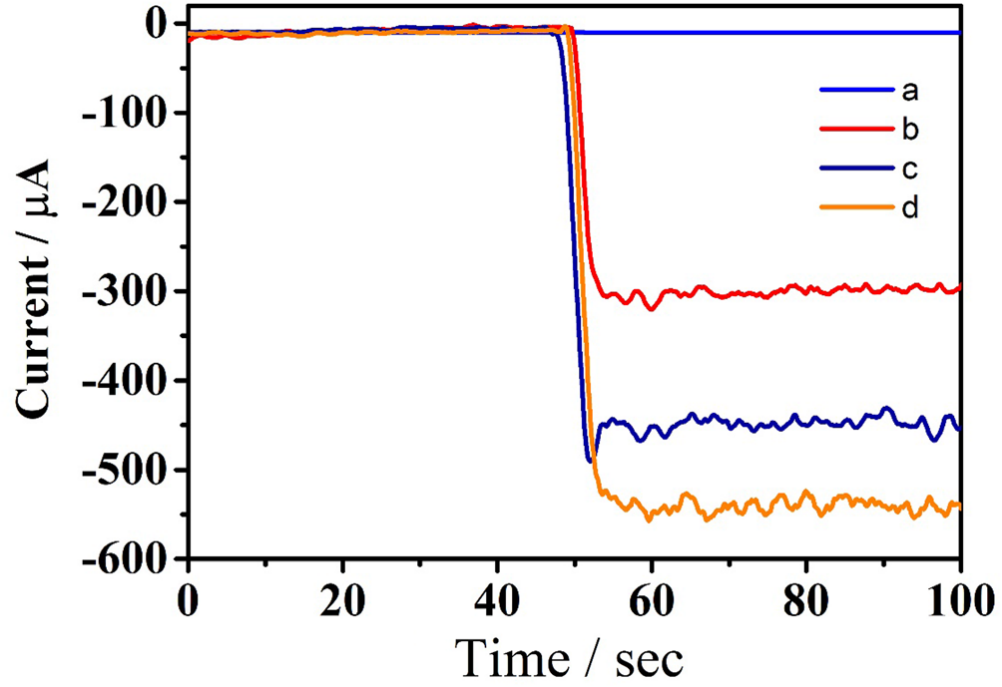
The i-t responses of different materials (i-t curve was recorded at − 0.4 V in pH 7.4 of PBS): (**a**) bare GCE; (**b**) Cu@L-Asp/Pt NPs; (**c**) Cu@L-Asp/Pd NPs; (**d**) Cu@L-Asp/Pt–Pd NPs.

Platinum nanoparticles and palladium nanoparticles can catalyze the decomposition of hydrogen peroxide(H_2_O_2_), so they are widely used as signal amplification materials in sensor field. Cu@L-Asp/Pd NPs, Cu@L-Asp/Pt NPs, Cu@L-Asp/Pd–Pt NPs could catalyze the decomposition of hydrogen peroxide and electron transfer occurs during that process so that the current change could be recorded. In this experiment we use the amperometric i-t curves to record the current values of different nanomaterials in PBS (PH = 7.4) adding the same concentration of H_2_O_2_ to verify the mechanism of our signal amplification strategy and the result is shown in Fig. 1. The bare GCE (curve a) didn’t have any electrocatalytic properties due to no electron transfer occurs at this stage. The current value increased to about 320 μA after Cu@L-Asp/Pt NPs (curve b) was coated onto the electrode. Compared with curve b, the current value is up to about 480 μA after the electrode was coated with Cu@L-Asp/Pd NPs. However, when we modified the electrode with Cu@L-Asp/Pt–Pd NPs, it was observed that the change value of current increased significantly. These results show that the catalytic performance of Cu@L-Asp/Pt–Pd NPs is better than that of Cu@L-Asp/Pt NPs and Cu@L-Asp/Pd NPs. Meanwhile, since horseradish peroxidase (HRP) is the traditional reagent to catalyze H_2_O_2_, we also compare the catalytic performance of Cu@L-Asp/Pt–Pd NPs and HRP, the result is shown in supplementary material Fig. S1. Therefore, we choose the Cu@L-Asp/Pd–Pt NPs nanocomposite as an optimal material for fabricating the proposed biosensor.

### Characterization of the Cu@L-Asp and Cu@L-Asp/Pd–Pt NPs

**Figure 2:**
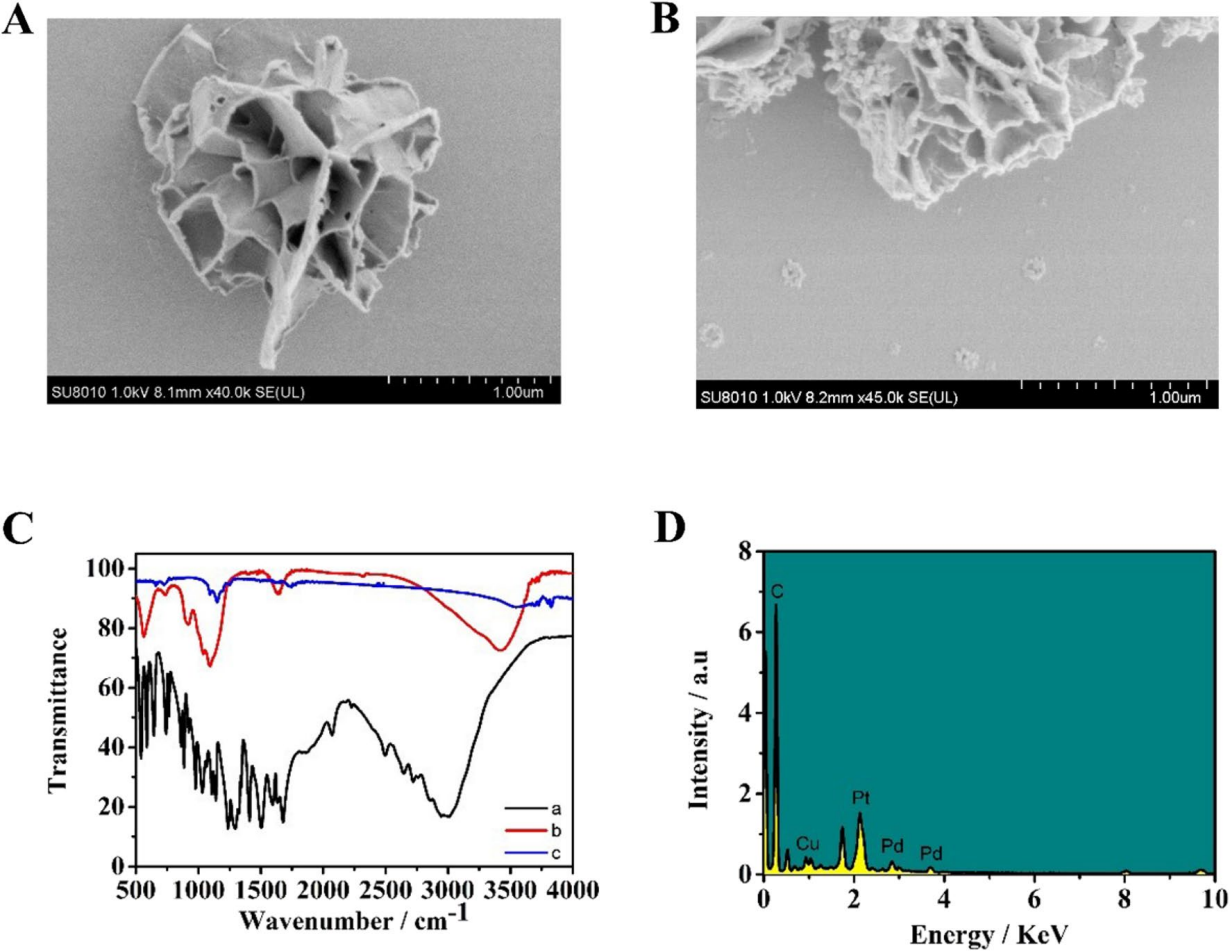
(**A**) jasmine-like Cu@L-Asp; (**B**) SEM images of Cu@L-Asp/Pd–Pt NPs; (**C**) The FT-IR spectra of (a) L-Asp; (b) Cu@L-Asp; (c) Cu@L-Asp/Pd–Pt NPs (**D**) The EDS spectra of Cu@L-Asp/Pd–Pt NPs.

We used SEM to investigate the shape and size of newly synthesized material. Figure 2A shows the overall morphology of Cu@L-Asp. The Cu@L-Asp exhibited smooth and crumpled surface with layered structure and showed jasmine-like structure with a diameter from 1 to 2 µm. After the Pd–Pt NPs got attached to Cu@L-Asp, a mass of protuberant dots was distributed on the surface of Cu@L-Asp hybrid nanoflower (Fig. 2B), which, suggests that Cu@L-Asp/Pd–Pt NPs nanocomposites have been successfully synthesized. In addition, the results of FT-IR also proved the successful synthesis of Cu@L-Asp and Cu@L-Asp/Pd–Pt NPs and the results are shown in Fig. 2C. In Cu@L-Asp/Pd–Pt NPs (curve b), the absorption peak of the asymmetric and symmetric COO^-^ at 1430 cm^−1^ and 1528 cm^−1^ disappear and the asymmetric stretching band of NH at 3047 cm^−1^ shifted to higher wavenumbers comparing with Cu@L-Asp (curve a). After palladium-platinum nanoparticles got attached to the Cu@L-Asp, the absorption peak of the asymmetric NH bond at 3467 cm^−1^ moved up to 3485 cm^−1^ (curve c), indicating the successful formation of Cu@L-Asp/Pd–Pt NPs nanocomposites. Meanwhile, elemental analysis of Cu@L-Asp/Pd–Pt NPs was carried out using EDS, and the results are shown in Fig. 2D and Table S1. XPS test was also performed to verify successful synthesis of the Cu@L-Asp/Pd–Pt NPs and the results are shown in Fig. S1.

### Electrochemical behavior of the modified electrodes

**Figure 3:**
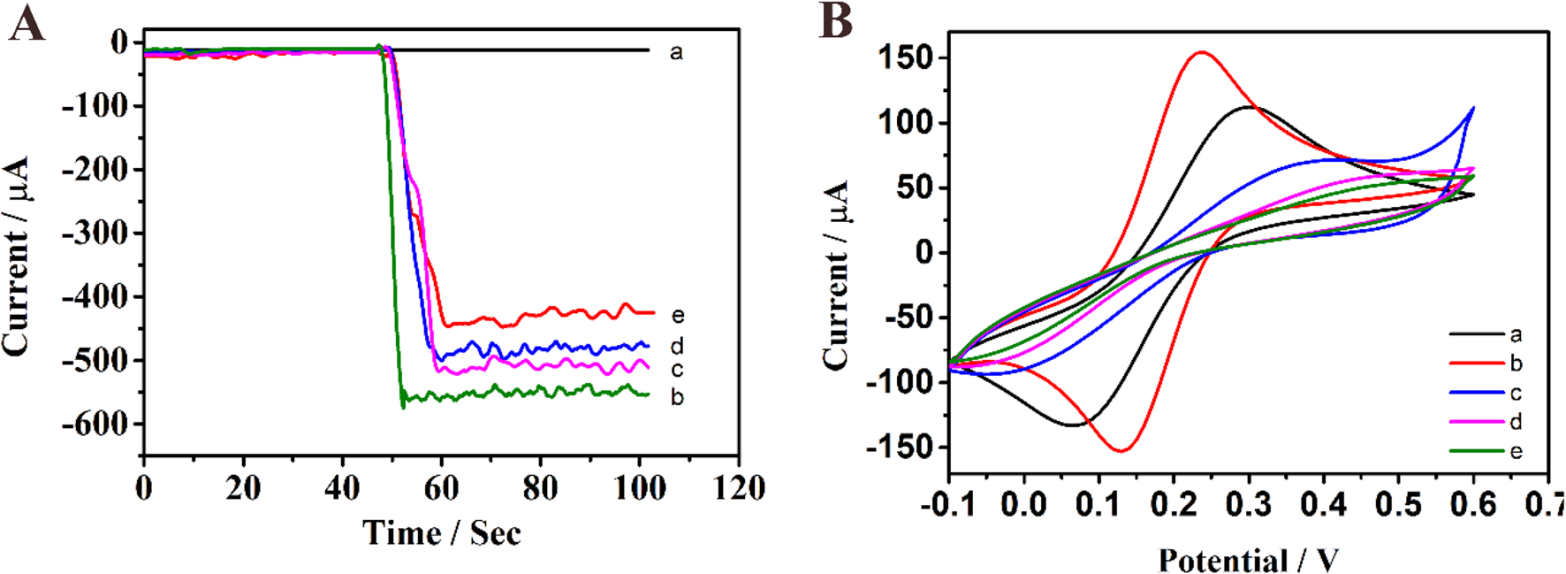
(**A**) The i-t curves of different coating stage (i-t curve was recorded at − 0.4 V in pH 7.4 of PBS) (**B**) CV characterization of electrodes at various stages of modification in a 5 mM [Fe(CN)6] ^3−/4−^ solution: (a) bare GCE; (b) Cu@L-Asp/Pd–Pt NPs/GCE; (c) anti-SPOP/Cu@L-Asp/Pd–Pt NPs/GCE; (d) BSA/anti-SPOP/Cu@L-Asp/Pd–Pt NPs/GCE; (e) target/BSA/anti-SPOP/Cu@L-Asp/Pd–Pt NPs/GCE.

In the process of electrode construction, we used i-t curves to characterize the successful implementation of each step. Figure 3A shows the electrode construction process verified by the i-t curve. Curve a corresponds to the bare GCE. Despite the presence of hydrogen peroxide, the current value is still almost zero for there is almost no decomposition of hydrogen peroxide at this time. Because the strong catalytic ability of the Cu @L-Asp/Pd–Pt NPs, when they were dropped onto the electrode, a strong current value could be observed (curve e). Curve d corresponds to the current value after dropping the antibody onto the electrode. The electron transfer process is hindered for the antibody is essentially a protein with poor electrical conductivity so that we can observe a significant decrease in the current value comparing with curve e. When the nonspecific sites were blocked with BSA, the current value declined further (curve c). Finally, due to the specific binding of antigen and antibody, when SPOP protein is added, the electron transfer process is further hindered, and the lower current value could be observed (curve b). At the same time, we also used CV curves to record the electrode construction process (Fig. 3B) at room temperature in a 5 mM[Fe(CN)6]^3−/4−^ solution containing 0.1 mol L^−1^ of KCL, and the scan rate is 50 mV s^−1^ ranging from − 0.1 to 0.6 V. Curve a shows a pair of distinct reversible redox peaks ,which represented the bare GCE. After the GCE was modified with Cu@L-Asp/Pd–Pt NPs, the peak current increased (curve b) due to the strong electrical conductivity of the Cu@L-Asp/Pd–Pt NPs nanocomposite. With the addition of antibody and BSA, the peak current value (curve c ,d) decreases gradually due to the obstruction of electron transfer and the decreased electrical conductivity. Finally, when we added the SPOP protein, the peak current value (curve e) declined to the lowest, indicating the successful construction of the label-free immunosensor.

### Optimization of the experimental conditions

**Figure 4:**
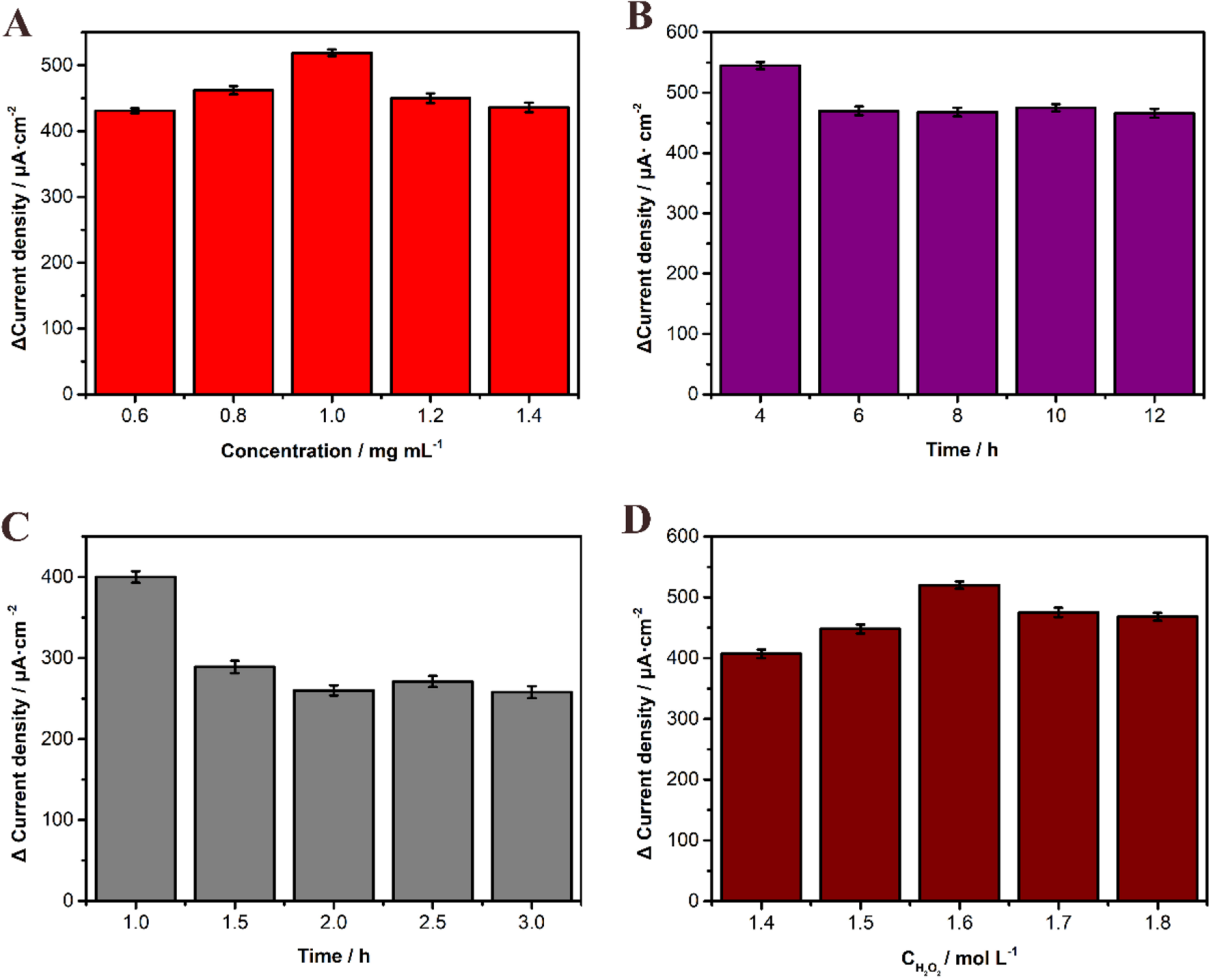
Optimization of the experimental conditions: (**A**) the concentration of Cu@L-Asp/Pd–Pt NPs nanocomposites; (**B**) the incubation time of anti-SPOP; (**C**) the reaction time of SPOP; (**D**) the concentration of H_2_O_2_.

In the process of detecting antigen concentration, many factors will affect the sensitivity of the proposed immunosensor. Therefore, we need to optimize the experimental conditions in the construction of the sensor. First, with other experimental conditions unchanged, the current value increases as the concentration of the Cu@L-Asp/Pd–Pt NPs nanocomposites increase, but when the concentration of the nanocomposites exceeds to 1 mg mL^−1^, instead, the current value decreases, so we choose 1 mg mL^−1^ as the optimal experimental concentration (Fig. 4A). The reason why excessive Cu@L-Asp/Pd–Pt NPs nanocomposites cause decreased current change is likely that superfluous L-Asp introduced into the reaction system makes the impedance increased and the advantage of the increased current caused by Pd–Pt NPs is counteracted. Secondly, antibody incubation time is another key factor affecting sensitivity of the sensor. When we gradually increase the incubation time to 4 h, the current value appears to decrease, so we choose 4 h as the best incubation time (Fig. 4B). Meanwhile, the reaction time between SPOP and anti-SPOP and the concentration of H_2_O_2_ has also taken into consideration (Fig. 4C,D) and finally we choose 1 h and 1.6 mol L^−1^as the optimal reaction time and concentration of H_2_O_2_.

### Analytical performance of the NMP-22 sensor

**Figure 5:**
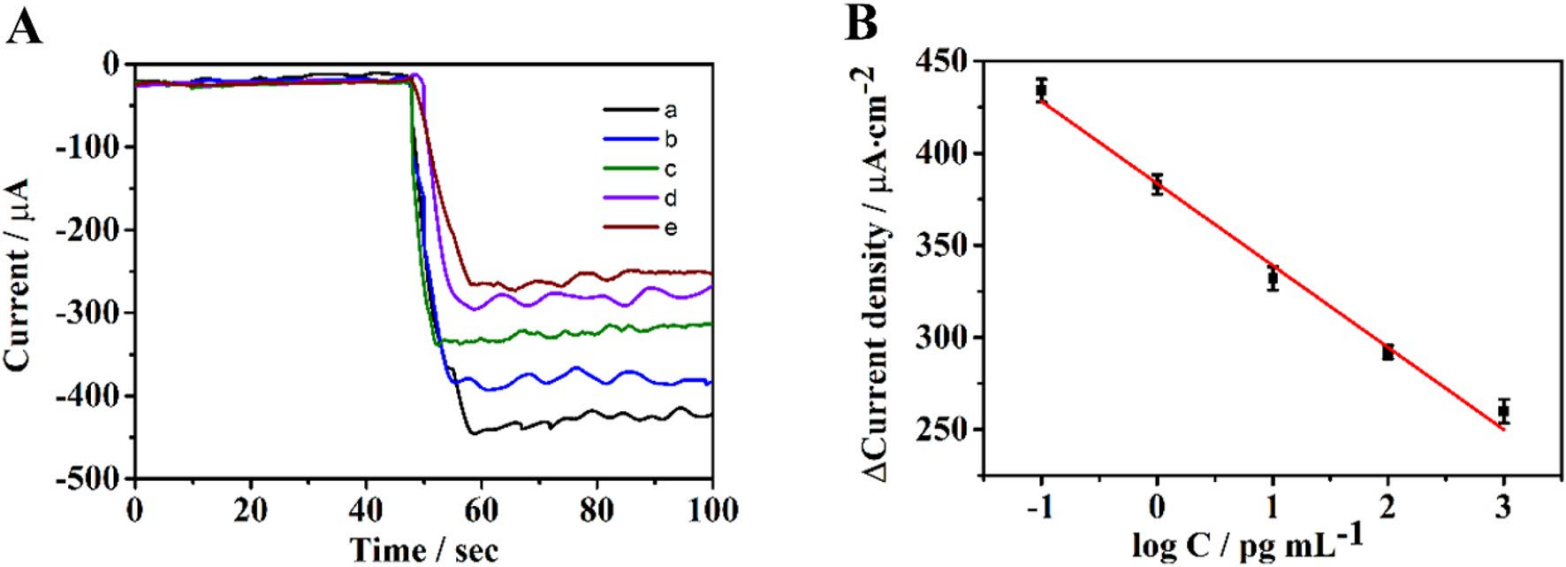
(**A**) The i-t response for the determination of different concentration of SPOP: (a) 0.1 pg mL^−1^; (b) 1 pg mL^−1^; (c) 10 pg mL^−1^; (d) 100 pg mL^−1^; (e) 1 ng mL^−1^. (**B**) The calibration plot of the SPOP sensor (n = 3).

Due to the complexity of the experimental environment, each concentration of the real sample is the average value of the parallel experiment repeated three times, however, this may still be different from the true value so that we use the least square method to calculate the linear regression equation and control the error within an acceptable range and the results are shown in the Fig. 5. When the concentration of SPOP was 0.1–1 ng mL^−1^, the current change value showed a good linear relationship with the logarithm value of the protein concentration, with a detection limit of 19 fg/mL (based on S/N = 3). The regression equation was Y = − 44.55logC_SPOP_ + 383.62(R^2^ = 0.9904), (where Y represents an amperometric i-t current increment, C_SPOP_ means the concentration of SPOP and R^2^ refers to the regression coefficient. The sensor constructed this time has a low detection limit and a wide detection range, which can be attributed to the excellent catalytic performance and large surface area of the first synthetic material. Additionally, our method is efficient and simple and can be operated easily.

### Stability, specificity and repeatability of the SPOP sensor

**Figure 6:**
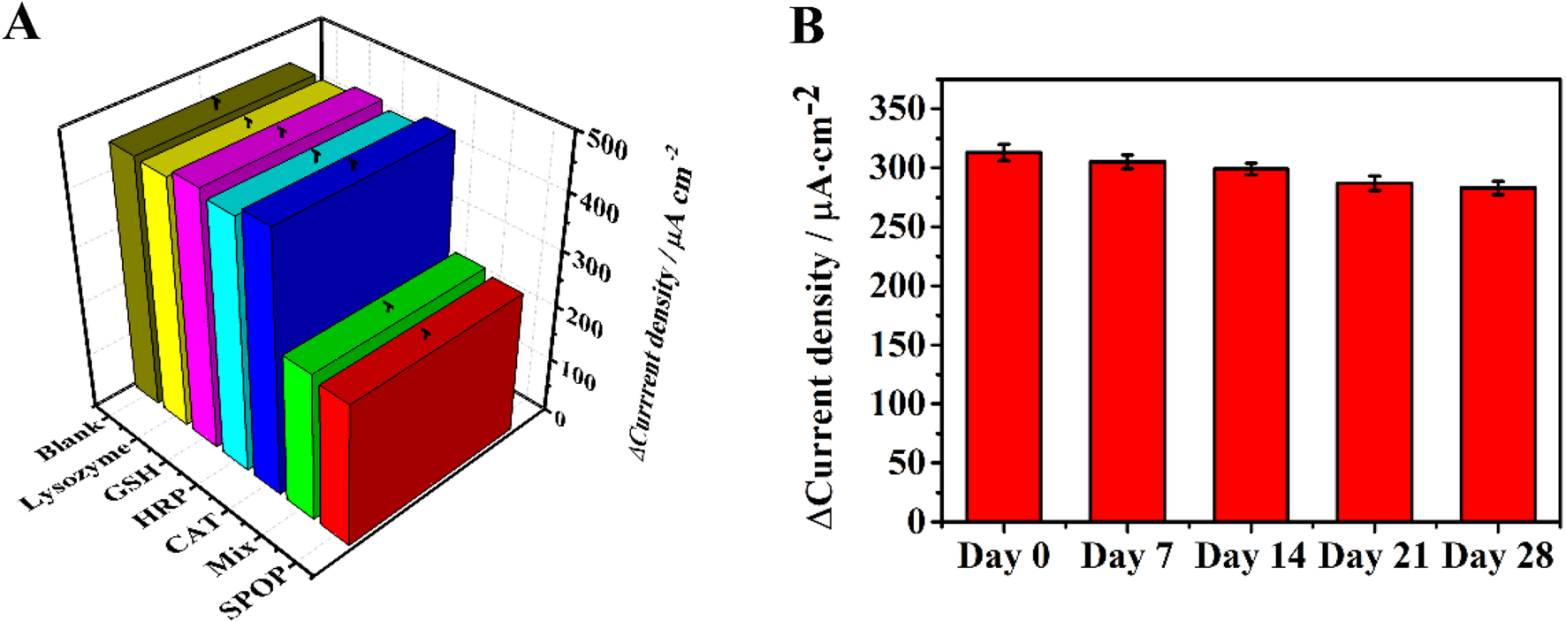
(**A**) Specificity of the immunosensor for SPOP (0.1 ng/mL), several competing 5 interfering substances (Lysozyme, GSH, HRP, CAT) (1 ng/mL), a mixture (Mix) of the interfering substances (1 ng/mL) and SPOP (0.1 ng mL^−1^) and zero analyte (Blank). (**B**) Stability of the immunosensor at 0 d, 7d, 14 d, 21 d, and 28 d. The concentration of SPOP was 10 pg mL^−1^.

To exhibit the proposed label-free immunosensor could specifically detect SPOP, we use different interfering substances including lysozyme asptathione, horseradish peroxidase, catalase. Under the same optimal experimental conditions, the specificity of the sensor was investigated using SPOP (0.1 ng mL^−1^) and interfering substances (1 ng mL^−1^). As shown in Fig. 6A, since the anti-SPOP antibody could only specifically bind SPOP, other interfering substances were removed from the electrode after washing procedure so that the different interfering substances and blank controls at the same concentration would hardly cause the current change, while when SPOP antigen at the same concentration was added and the mixed group was added, the current value decreased significantly, which, suggests that the selectivity of the proposed immunosensor was acceptable. To assess the stability of the sensor, it was conserved at 4 °C in a fridge. After storage for 28 days, the final current change recorded by the i-t curves was 91.5% of its initial current value (Fig. 6B) probably because the decomposition of the Cu@L-Asp/Pd–Pt NPs. The above results revealed that the proposed immunosensor exhibits an acceptable stability.

The repeatability of the immunosensor was estimated by the measurement of the same concentration of SPOP (10 pg mL^−1^) at room temperature using five different electrodes. The relative standard deviation (RSD) of the sensor was 0.52%, suggesting it has good repeatability. The result is shown in supplementary materials.

### Application of the immunosensor in real sample

In order to investigate the potential applications of the proposed sensors for detecting real samples, the different concentrations of SPOP in human IOSE80 cell line was determined by applying the immunosensor to the human IOSE80 cell line and the results were compared with the ELISA method (Table 1, Fig. S3). It showed that the RSD range of the established immunosensor for SPOP determination was 2.37% ~ 3.99%, the recovery rates were between 98.50% and 100.99%, which suggests that the results of using immunosensor were better than that of ELISA and this method could be used for detecting the SPOP in human cell samples.

**Table 1:** Comparison of determination for SPOP levels by two methods.

Sample	Measured (pg mL^−1^)	Added (pg mL^−1^)	ELISA			This work		
			Found (pg mL^−1^)	RSD (%)	Recovery (%)	Found (pg mL^−1^)	RSD (%)	Recovery (%)
1	100.21	100	208.34	6.81	104.89	202.21	3.26	100.99
2	100.34	200	304.57	6.93	101.39	302.114	3.99	100.58
3	99.87	300	378.21	6.72	94.58	393.89	2.37	98.50
4	100.44	400	536.57	7.13	107.22	502.09	2.22	100.33
5	100.19	500	660.21	6.88	110.01	608.83	2.59	101.44
6	99.33	600	768.42	7.07	109.88	699.19	3.66	99.98

## Conclusions

In this work, we first synthesize three-dimensional jasmine-like Cu@L-Asp hybrid nanoflowers with large surface area in order to load platinum and palladium nanoparticles as signal amplification materials for detecting SPOP, the results showed that the proposed Cu@L-Asp/Pd–Pt NPs has good performance of catalyzing decomposition of hydrogen peroxide. Furthermore, other methods used for detecting SPOP have also been compared with this work in Table S3 and the result showed that the proposed method is simple and efficient with acceptable specificity, stability and good repeatability, offering a new way for detecting SPOP. However, we found in the experiment that no matter how we adjust the temperature and PH value, the sensor can only detect a single substance and cannot be recycled. Therefore, we will try to overcome the above shortcomings in future research and design a simpler and more efficient sensor for detecting SPOP or other intracellular macromolecular substances.

## Supplementary Information

Supplementary Information.
