# Supplementary material for: The establishment of an immunosensor for the detection of SPOP
Source: Sci Rep. 2021 Jun 15;11:12571. doi: 10.1038/s41598-021-91944-3 (PMC8206368; doi:10.1038/s41598-021-91944-3)
Supplement: Supplementary file 1 — Supplementary Information. [file 41598_2021_91944_MOESM1_ESM.docx]

**The establishment of an immunosensor for the detection of SPOP**

Song Yue^1#^, Kexin Sun^2#^, Siyuan Li^1^, Yi Liu^1^, Qihao Zhu^1^, Yiyu Chen^1^, Dong Yuan^1^, Tao Wen^1^, Mingjian Ge^3^* and Qiubo Yu^1^*.

1 Institute of Life Science, Chongqing Medical University, Chongqing 400016, P.R. China.

2 Department of Ophthalmology, The First Affiliated Hospital of Chongqing Medical University, Chongqing Key Laboratory of Ophthalmology, Chongqing Eye Institute, Chongqing, 400016, P.R. China.

3 Department of thoracic surgery, The First Affiliated Hospital of Chongqing Medical University, Chongqing, 400016, P.R. China.

Song Yue^1#^, Kexin Sun^2#^ contribute equally to this work

Mingjian Ge^3^* and Qiubo Yu^1^* are joint corresponding authors

Phone: (86)23 -68815186

Fax: (86) 23-68865186

E-mail: yqb76712@gmail.com

Full address: Chongqing Medical University, 1 Yi Xue Yuan Road, Chongqing 400016, P. R. China.

**S1.** **Catalytic performance between HRP and** **Cu@L-Asp Pt-Pd NPs nanoflowers**

**
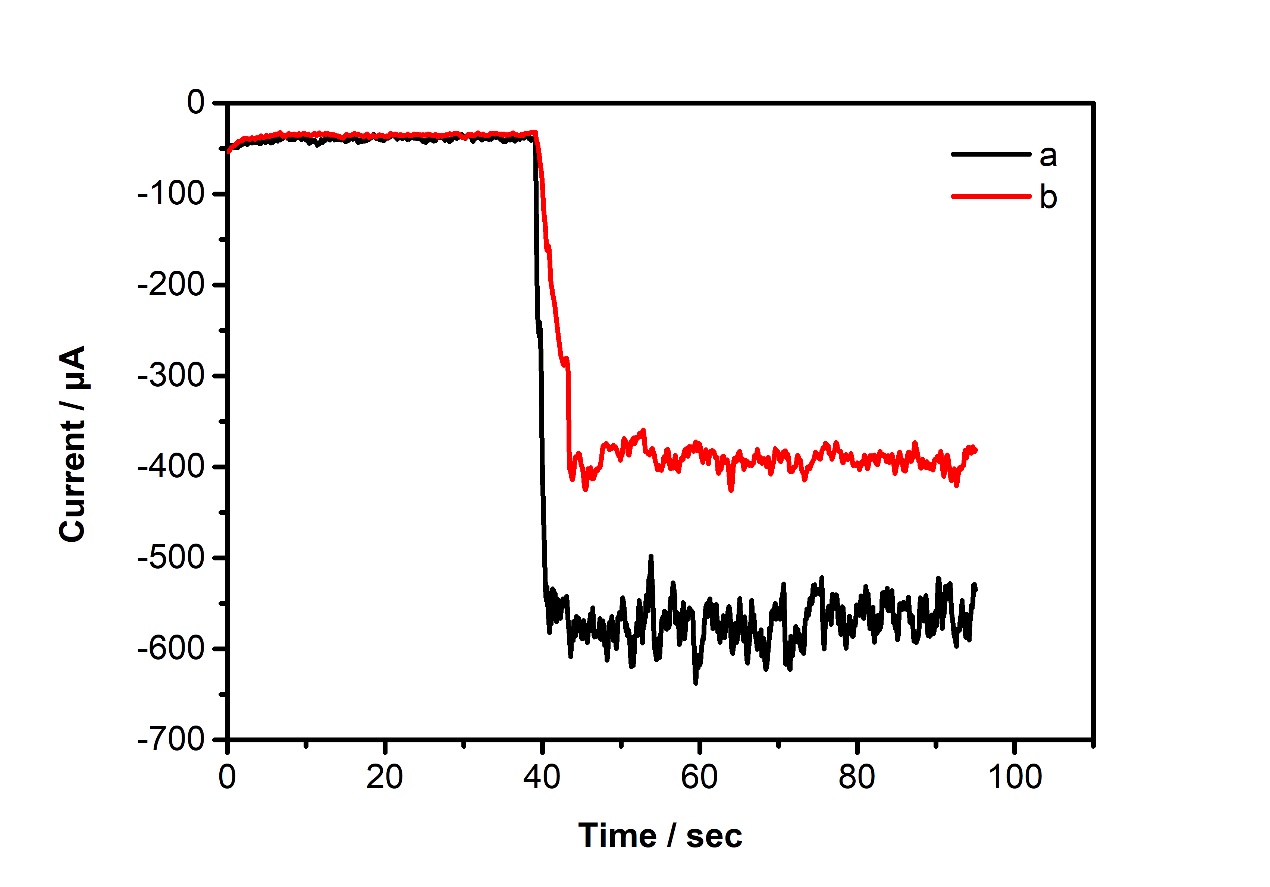
**

**Fig S1 Catalytic performance between Cu@L-Asp Pt-Pd NPs(a) and HRP(b)**

**S2. Synthesis of Pd NPs and Pt NPs**

The synthetic methods of Pd NPs and Pt NPs is according to the literature[[1](#_ENREF_1)]. 10 mL of NaBH_4_ solution was added into solution consists of 170 µl of 5% Na_2_PdCl_4_ and 10 mL of ultrapure water with stirring at 400 rpm for 1h. After that, use ultrapure water and ethanol for washing three times, respectively. Finally, the resulting preparation was dispersed in the 2 mL of ultrapure water for further use. Pt NPs were synthesized using a similar method except that 170 µl of Na_2_PdCl_4_(5%) was replaced with 300 µl of H_2_PtCl_6_(5%).

**S3. XPS analysis of the Cu@L-Glu/Pd-Pt NPs**


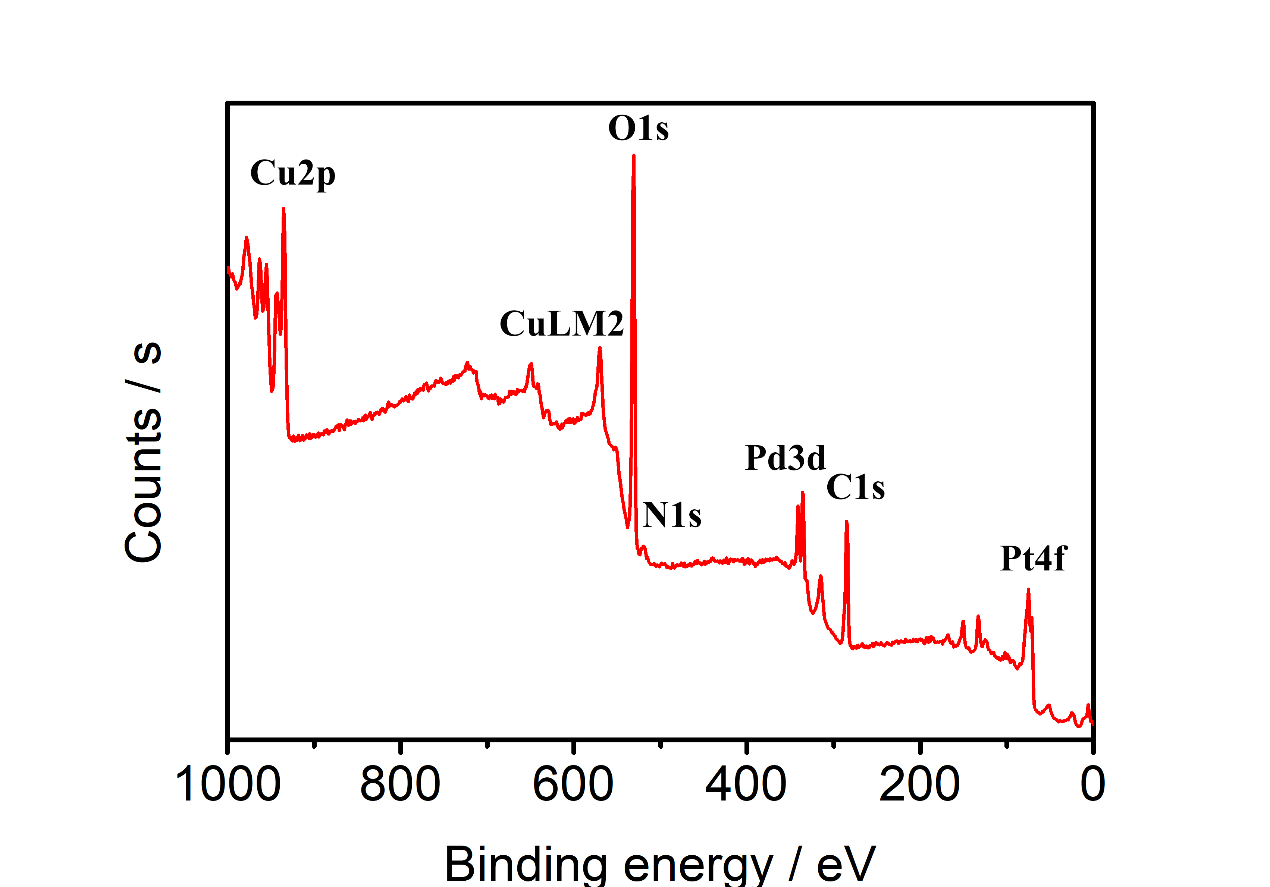


**Fig. S2. XPS analysis of the Cu@L-Glu/Pd-Pt NPs**

**S4. The repeatability of the proposed immunosensor**


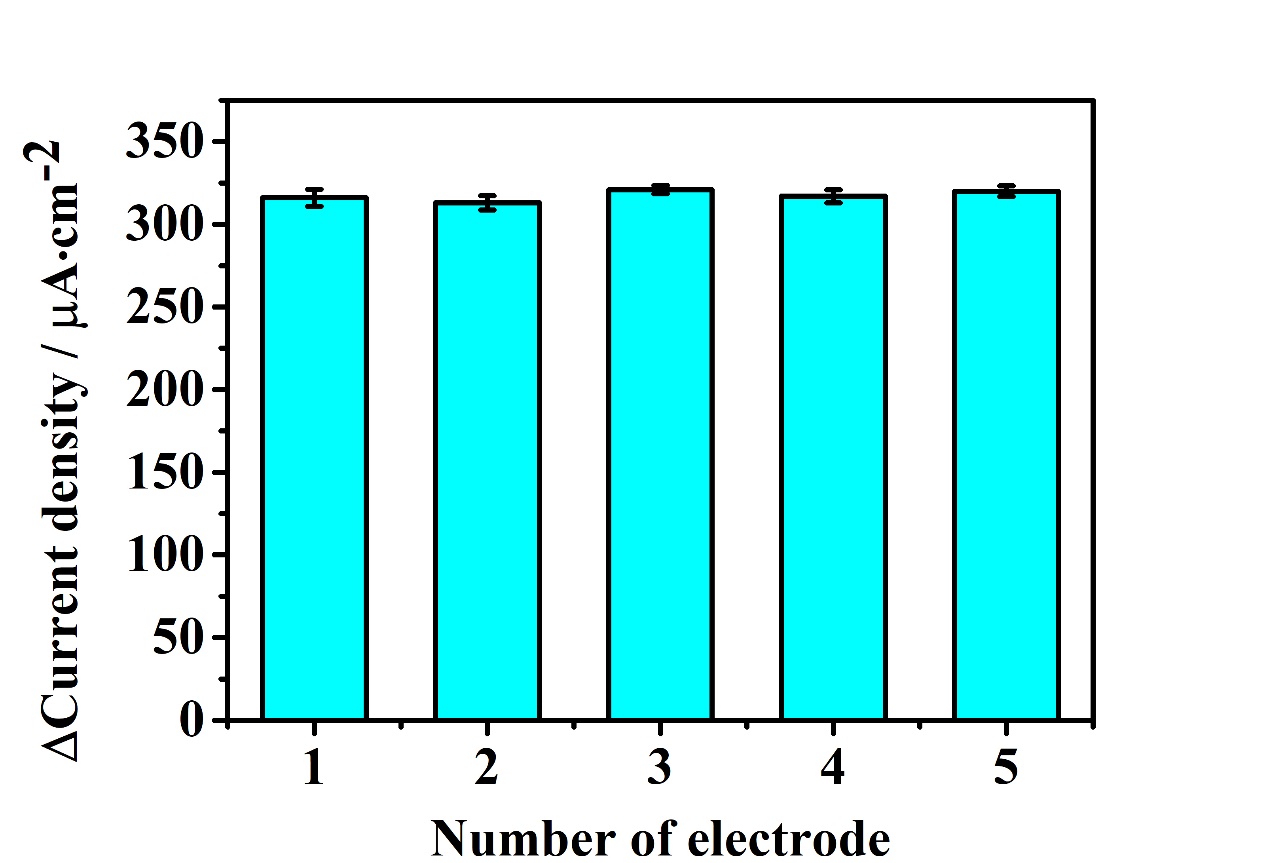


Fig. S3. Repeatability results for five groups of identical electrodes(n=3) incubated with the same concentration of SPOP(10 pg mL^-1^)

**Table S1. EDS analysis of the Cu@L-Glu/Pd-Pt NPs**

| Element | Line Type | Apparent Concentration | k Ratio | Wt% | Wt% Sigma | Standard Label | Factory Standard |
| --- | --- | --- | --- | --- | --- | --- | --- |
| C | K series | 29.38 | 0.29383 | 80.77 | 1.16 | C Vit | Yes |
| Cu | K series | 2.47 | 0.02473 | 5.30 | 0.47 | Cu | Yes |
| Pd | L series | 3.85 | 0.03849 | 7.90 | 0.52 | Pd | Yes |
| Pt | M series | 3.12 | 0.03119 | 6.03 | 0.82 | Pt | Yes |
| Total: |  |  |  | 100.00 |  |  |  |

**Table S2. Analytical performance compared with previous reported sensors.**

| Signal amplification strategy | Target | Linear range | LOD | Reference |
| --- | --- | --- | --- | --- |
| SA-β-Gal-CaHPO4 nanoflower | AFP | 0.1ng mL^-1^-10 ng mL^-1^ | 0.17 ng mL^-1^ | 1 |
| SA-HRP-Cu3(PO4)2 nanoflower | AFP | 0.1ng mL^-1^-50 ng mL^-1^ | 78.00 pg mL^-1^ | 2 |
| Pd–Pt bimetallic nanocrystals | hTPA | 0.005ng mL^-1^-15 ng mL^-1^ | 1.20 pg mL^-1^ | 3 |
| Metal–organic frameworks supported surface–imprinted nanoparticles | Metolcarb | 0.1mg L ^-1^-0.9 mg L ^‑1^ | 0.07 mg L^-1^ | 4 |
| ZnO@MIP | NMP-22 | 128–588 ng/mL ^-1^ | - | 5 |
| Fe3O4/g-C3N4/HKUST-1 | Ochratoxin A | 5.0 ng mL^-1^-160 ng mL^-1^ | 2.57 ng mL^-1^ | 6 |
| Graphene oxide nanoribbons (GONRs) | DA | 15.32 ng mL^-1^-1302 ng mL^-1^ | 3.67 ng mL^-1^ | 7 |
| Au@Ag NPs-GOx | CEA | 100 fg mL^-1^-5 pg mL^-1^ | 0.02 pg mL^-1^ | 8 |
| N, S-GQDs@Au-PANI | CEA | 0.5ng mL^-1^-1000 ng mL^-1^ | 0.01 pg mL^-1^ | 9 |
| Guanine-assembled graphene nanoribbons (G-GNRs) and magnetic beads | Brevetoxin B | 0.1ng mL^-1^-50 ng mL^-1^ | 1.00 pg mL^-1^ | 10 |
| Pt-pd/Cu@L-Asp | SPOP | 0.1 pg mL^-1^-1 ng mL^-1^ | 19.00 fg mL^-1^ | This work |

**References**

1. Yu, L. *et al.* Self-assembled protein-enzyme nanoflower-based fluorescent sensing for protein biomarker. *Analytical and Bioanalytical Chemistry* **16**, 1798-1302 (2018).
2. Yu, L. *et al*. The preparation of dual-functional hybrid nanoflower and its application in the ultrasensitive detection of disease-related biomarker. *Biosensors and Bioelectronics* **92**, 68-73 (2017).
3. Yao, W. *et al.* A sandwich-type immunosensor using Pd–Pt nanocrystals as labels for sensitive detection of human tissue polypeptide antigen. *Nanotechnology* **25**, 55-78 (2014).
4. Kun, Q. *et al*. Metal–Organic Frameworks Supported Surface– Imprinted Nanoparticles for the Sensitive Detection of Metolcarb. *Biosensors and Bioelectronics* **79**, 359-363 (2016).
5. Mei, L. *et al.* Electrochemical sensing of nuclear matrix protein 22 in urine with molecularly imprinted poly(ethylene-co-vinyl alcohol) coated zinc oxide nanorod arrays for clinical studies of bladder cancer diagnosis. *Biosensors and Bioelectronics* **79**, 789-795 (2016)
6. Yu, Y. *et al*. A novel label-free electrochemical immunosensor based on functionalized nitrogen-doped graphene quantum dots for carcinoembryonic antigen detection *Biosensors and Bioelectronics* **90**, 31-38 (2018).
7. Chia, S. *et al*. Synthesis of short graphene oxide nanoribbons for improved biomarker detection of Parkinson's disease *Biosensors and Bioelectronics* **67**, 327-333 (2015).
8. Sattar A,. *et al*. Gold and silver bio/nano-hybrids-based electrochemical immunosensor for ultrasensitive detection of carcinoembryonic antigen disease Biosensors and Bioelectronics **20**, 147-153 (2019).
9. Akhilesh BabuGanganboina1 & Ruey-An Doong Graphene Quantum Dots Decorated Gold-Polyaniline Nanowire for Impedimetric Detection of Carcinoembryonic Antigen *Scientific Reports* **91**, 7214 (2019).
10. Juan, T. *et al*. Magneto-controlled electrochemical immunoassay of brevetoxin B in seafood based on guanine-functionalized graphene nanoribbons *Biosensors and Bioelectronics* **38**, 86-93 (2012).
